# The Role of Multimodality Imaging in Left-Sided Prosthetic Valve Dysfunction

**DOI:** 10.3390/jcdd9010012

**Published:** 2022-01-04

**Authors:** Manuela Muratori, Laura Fusini, Maria Elisabetta Mancini, Gloria Tamborini, Sarah Ghulam Ali, Paola Gripari, Marco Doldi, Antonio Frappampina, Giovanni Teruzzi, Gianluca Pontone, Piero Montorsi, Mauro Pepi

**Affiliations:** 1 Centro Cardiologico Monzino IRCCS, 20138 Milan, Italy; manuela.muratori@cardiologicomonzino.it (M.M.); maria.mancini@cardiologicomonzino.it (M.E.M.); gloria.tamborini@cardiologicomonzino.it (G.T.); sarah.ghulamali@cardiologicomonzino.it (S.G.A.); paola.gripari@cardiologicomonzino.it (P.G.); doldi.mrc@gmail.com (M.D.); afrappampina@gmail.com (A.F.); giovanni.teruzzi@cardiologicomonzino.it (G.T.); gianluca.pontone@ccfm.it (G.P.); piero.montorsi@cardiologicomonzino.it (P.M.); mauro.pepi@cardiologicomonzino.it (M.P.); 2 Department of Electronics, Information and Bioengineering, Politecnico di Milano, 20133 Milan, Italy; 3 Department of Clinical Sciences and Community Health, Cardiovascular Section, University of Milan, 20122 Milan, Italy

**Keywords:** prosthetic valve dysfunction, structural valve degeneration, endocarditis, echocardiography, fluoroscopy, computed tomography

## Abstract

Prosthetic valve (PV) dysfunction (PVD) is a complication of mechanical or biological PV. Etiologic mechanisms associated with PVD include fibrotic pannus ingrowth, thrombosis, structural valve degeneration, and endocarditis resulting in different grades of obstruction and/or regurgitation. PVD can be life threatening and often challenging to diagnose due to the similarities between the clinical presentations of different causes. Nevertheless, identifying the cause of PVD is critical to treatment administration (thrombolysis, surgery, or percutaneous procedure). In this report, we review the role of multimodality imaging in the diagnosis of PVD. Specifically, this review discusses the characteristics of advanced imaging modalities underlying the importance of an integrated approach including 2D/3D transthoracic and transesophageal echocardiography, fluoroscopy, and computed tomography. In this scenario, it is critical to understand the strengths and weaknesses of each modality according to the suspected cause of PVD. In conclusion, for patients with suspected or known PVD, this stepwise imaging approach may lead to a simplified, more rapid, accurate and specific workflow and management.

## 1. Introduction

Prosthetic valve (PV) dysfunction (PVD) is relatively rare yet potentially life-threatening. Although often challenging, establishing the exact cause of PVD is essential to determine the appropriate treatment strategy. We all know that in clinical practice, the assessment of PV morphology and function is primarily assessed with 2D/3D transthoracic (TTE) and transesophageal echocardiography (TEE). Indeed, echocardiography is the first step examination and may be performed in every setting including outpatient laboratories, emergency departments and operating theatres. More importantly, both TTE and TEE may be carried out in cases of stable or unstable hemodynamic conditions. Despite echocardiography often leading to a conclusive diagnosis, fluoroscopy, multidetector computed tomography (CT), cardiac magnetic resonance (CMR), and, to a lesser extent, nuclear imaging are all complementary tools for the diagnosis and management of PV complications.

In recent decades, PV design and models have changed greatly and several new biological and mechanical PV types were introduced in surgical and percutaneous procedures. Despite the improved hemodynamics and the increased durability, all PV are prone to dysfunction. The main causes of PVD leading to surgical PV reoperation are: thrombus/pannus prosthetic obstruction; paravalvular leak (PVL); bioprosthetic structural valve degeneration (SVD); and PV endocarditis (PVE).

Current guidelines have highlighted the value of TTE Doppler parameters for PVD detection [1]. Multiple cross-sectional and off-axis views are required to visualize the mobility of PV leaflet (s). Color Doppler is used for screening and evaluating the degree of intra and/or paraprosthetic regurgitation. Doppler-derived parameters in prosthetic mitral valve evaluation include: early peak mitral velocity (E wave); mean transprosthetic gradient (∆P_mean_); pressure half time (PHT); and Doppler velocity index (DVI). Doppler-derived parameters in prosthetic aortic valve evaluation include: peak transprosthetic velocity (Vmax); peak and mean transprosthetic gradients (∆P_peak_ and ∆P_mean_); DVI; effective prosthetic orifice area (EOA); acceleration time (AT); ejection time (ET); and the ratio AT/ET. TEE is the diagnostic tool which best defines the causes of PVD and helps in guiding therapy, risk stratification and follow-up. TEE must include 2D and 3D conventional and off-axis views and allows the identification of thrombus, pannus ingrowth, paraprosthetic regurgitation and endocardial vegetations/abscess and fistulae.

The present review discusses recommendations for the use of multimodality imaging in the assessment of PVD.

## 2. Prosthetic Valve Obstruction

The most common causes of obstruction are valve thrombosis and pannus ingrowth. Thrombosis is more likely to occur early after PV implantation in a setting of inadequate anticoagulation and it is more common with mechanical prostheses [2,3]. Nevertheless, it may also occur with biological prostheses and it may develop long after valve implantation [4]. This underscores the importance of serial imaging follow-up. It must be noted that, in general, thrombosis of biological PV in the mitral position is usually a subacute or chronic process, rather than an acute phenomenon.

Pannus ingrowth, which is more frequent in the context of PV in the aortic position, depends by fibroblast proliferation, collagen deposition and neoangiogenesis. Unlike thrombosis, pannus is more likely to occur long after valve replacement, independently from anticoagulation level, and it is generally associated with a progressive onset of the symptoms [5].

### 2.1. Prosthetic Mitral Valve Obstruction

**Figure 1:**
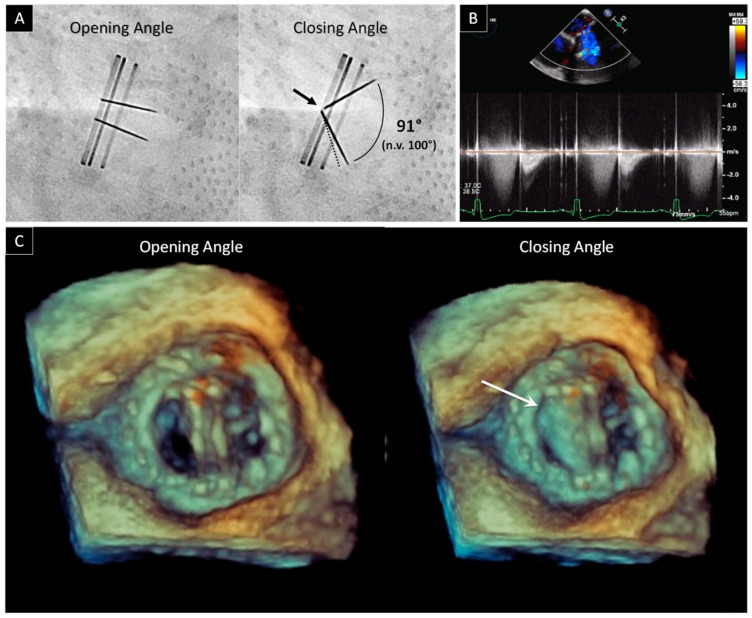
Bileaflet mechanical mitral valve obstruction. (**A**) Fluoroscopic evaluation of normal opening and abnormal closing angles in a patient with prosthetic mitral valve dysfunction. The dotted line refers to the normal leaflet closure. The abnormal leaflet closure was confirmed by the lack of leaflets contact at the hinge area (black arrow). (**B**) Holosystolic regurgitation at continuous wave Doppler by TEE. (**C**) 3D view of prosthetic mitral valve from atrial side. During systole, only one leaflet closes (white arrow).

The presentation of prosthetic obstruction in the mitral position can vary from mild dyspnea to severe acute pulmonary edema. Timely diagnosis through evaluation with multimodality imaging is key, especially in the case of mechanical mitral PV, where a rapid deterioration can occur in the case of thrombus causing leaflet opening malfunctioning (Figure 1).

The first step is to perform a TTE targeted to the anatomy of the biological PV or to the leaflet(s) movement of the mechanical PV [6]. Furthermore, it is important to have TTE-Doppler performed using both flow-dependent parameters (ΔP_mean_ and E wave) as well as flow-independent parameters, such as DVI. This latter provides a measure of PV function independent of cardiac output variation. The suggested approach increases the diagnostic insight of TTE in suspected PVD [1].

The second step is to perform fluoroscopy, a low-cost, noninvasive imaging technique, readily available in most centers that can be performed rapidly, particularly in unstable patients, for detecting stuck valves. Fluoroscopy allows direct visualization of the prosthetic disks and the measuring of the opening and closing angles [7]. In particular, three studies of Montorsi et al. [7,8,9] showed the importance of fluoroscopy. In the first two, it is shown how fluoroscopy effectively leads to the identification of the so-called “Doppler-silent” thrombosis (i.e., mitral bileaflet PV with the beginning of dysfunction, characterized by abnormal leaflet motion but a normal ΔP_mean_ at rest). The third study underlines the utility of fluoroscopy to ensure adequate follow-up in patients undergoing thrombolysis. Fluoroscopy also has limitations: in fact it is not useful in distinguishing pannus from thrombus since neither pannus nor thrombus can be identified fluoroscopically. Therefore, the combined evaluation by TTE and fluoroscopy is essential in the diagnostic workup for the mechanical mitral valve obstruction. In this regard, we recently published data concerning the incremental value of the combined approach (TTE plus fluoroscopy) over TTE or fluoroscopy alone in patients with suspicions of PVD [10].

TEE is the next mandatory step needed to confirm diagnosis and to identify the best therapy. TEE makes it possible to identify prosthetic thrombus, defined as irregular shaped mobile masses with low echogenicity, and to measure the dimensions of clots (<> 0.8 cm^2^). The clot size determines whether to go with either urgent treatment, with slow-infusion of low-dose fibrinolytic therapy, or with emergency surgery [11]. TEE also provides direct imaging of the thrombus, both in the body of the appendage and in the left atrium, both of which cannot usually be detected by TTE. According to current guidelines, the presence of a left atrial thrombus is an element that favors surgery over fibrinolysis and, therefore, should be ruled out before fibrinolysis [11]. TEE is also essential in pannus identification. Pannus, defined as a mass with lower area and lower density than thrombus, can produce an ineffective or partially effective fibrinolysis. This is commonly located on the ventricular side of the mitral prostheses and typically induces a steady or intermittent opening and closing of the leaflets. Pannus and thrombosis may also occur simultaneously, and a component of thrombus formation is often observed in concert with fibrotic pannus ingrowth. TEE also allows for the identification of a particular category of obstructions that may occur with endocarditis. Indeed, vegetations due to PVE present as irregular shaped mobile masses with low echogenicity and may interfere with valve flow. Like thrombi, vegetations tend to start in the valve ring area and then spread to the leaflets, although often without opening and closing angles malfunctioning. As it may be difficult to differentiate between vegetations and thrombi on imaging, both should be considered in the clinical context.

Since 2007, real-time 3D TEE greatly expanded the functionalities of TEE, becoming a milestone in the era of cardiovascular imaging for both native and prosthetic mitral valves [12]. This is an excellent tool for getting spatial information from cardiac structures and visualizing cardiac pathologies in real-time. In a normal mechanical, bileaflet prosthesis, the “en face” perspective allows for the definition of two closed, semicircular discs in systole and three orifices (a small slit-like central orifice and two larger semicircular orifices) in diastole. In a normal biological PV, the three leaflets can be visualized in diastole and in systole. Due to the lack of major reverberations and shadowing with bioprosthetic valves, stents and leaflets of biological PV can also be imaged from the ventricular perspective. 3D TEE also has the ability to section the echogenic mass and visualizes it from multiple angles, thus enabling the identification of the presence, extension and localization of thrombus, pannus or vegetation.

The last diagnostic step is CT, which can be used as a complementary diagnostic method for a definitive diagnosis in inconclusive cases. In particular, CT might be helpful in patients with double left-sided mechanical valves (acoustic shadowing can in fact occur also during TEE making the interpretation difficult), in the identification of pannus and in the assessment of abscesses in the context of PVE [13]. Moreover, CT may also show dynamic alterations of disc motion, as an alternative to fluoroscopy. What has been written so far also applies to bioprosthetic mitral valves, with the notable exception of fluoroscopy, which of course is not useful in this context while once again 2D-3D TEE and CT are mandatory diagnostic tools.

### 2.2. Prosthetic Aortic Valve Obstruction

**Figure 2:**
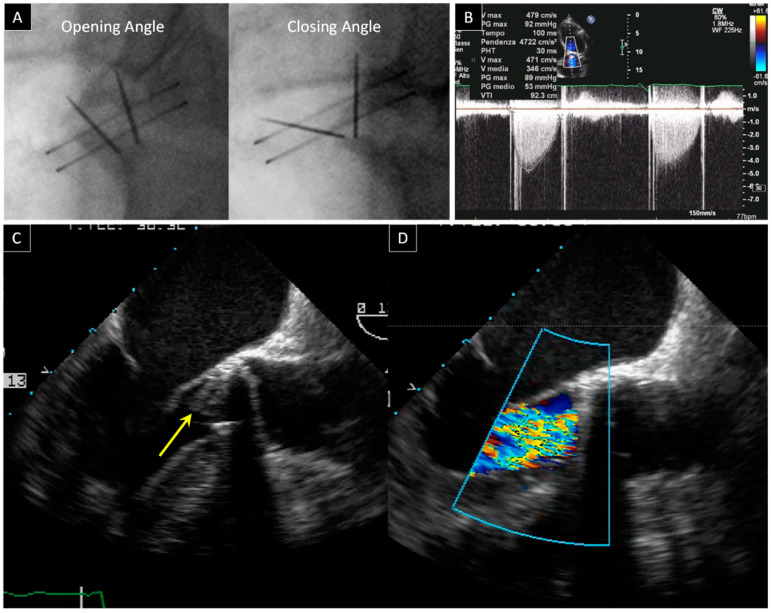
Bileaflet mechanical aortic valve obstruction. (**A**) Fluoroscopy shows normal opening and abnormal closing angles in a patient with prosthetic aortic valve dysfunction. (**B**) Flow acceleration of the anterograde flow is identified with color flow imaging from the TTE apical approach and it is associated with high transprosthetic gradients at continuous wave Doppler (ΔP_mean_ 53 mmHg). (**C**) 2D TEE from a 120° view reveals a thrombotic hyperechogenic mass on the ventricular side of the prosthesis (arrow). (**D**) Severe intraprosthetic regurgitation as assessed by 2D TEE.

Whilst the risk of thrombosis and thromboembolic events is higher in mitral versus aortic PV, recent data from CT imaging studies show that mechanical and biological prosthetic aortic valve thrombosis and pannus are relatively common phenomena that might be associated with an increased risk of stroke [14,15] (Figure 2).

TTE is the first line of examination with the important caveat of not being able to show, in most cases, leaflet’s motion in aortic mechanical bileaflet PVs, which are the most frequently implanted valves in the world [6]. TTE-Doppler evaluation must be performed using both flow-dependent parameters (V_max_, ΔP_peak_, ΔP_mean_, EOA) as well as flow-independent parameters (DVI, AT, ET, and AT/ET). Together they provide a measure of PV function independent of cardiac output variation. The combined use of flow dependent and flow independent parameters allows a better discrimination between normal PV, prosthesis-patient-mismatch phenomenon, and PV obstruction [16,17].

The second diagnostic step is to perform fluoroscopy, which remains key for the study of the bileaflets aortic PV despite as many as 40% of patients with pannus showing normal opening and closing angles at fluoroscopy [18]. As described in a recent biomedical engineering in vitro study [19], sub-prosthetic pannus only altered disc motion when the circumferential involvement of the structure came close to 360°, while an eccentric pannus did not induce leaflet restriction until an involvement angle of 180°. This explains the failure of fluoroscopy.

The aortic prosthesis is smaller and further away from the transducer than the mitral PV. As a result, the 2D and 3D TEE assessment of the PV in the aortic position is less effective than that of the PV in a mitral position. Nevertheless, TEE remains essential to confirm the diagnosis of thrombosis or endocarditis, to quantify the degree of obstruction and to guide towards the best therapy. Still, TEE may also fail to identify the presence of pannus growing on the ventricular surface of the aortic PV, as pannus can be easily masked by the prosthetic sewing ring.

Furthermore, in patients with inconclusive TTE and TEE findings, CT, thanks to its excellent spatial resolution, can provide an accurate evaluation of the PV structure and functional status. The use of cardiac CT for the assessment of possible prosthetic aortic valve dysfunction has risen rapidly over the past 10 years. One important reason for this is that CT can differentiate between thrombus and fibrotic pannus ingrowth based on Hounsfield units, with thrombus having lower attenuation than fibrotic pannus ingrowth [20]. CT can also help in the identification of premature bioprosthetic leaflet degeneration, in the assessment of mechanical leaflet motion and in the assessment of aortic root abscess formation [14,21]. For all these reasons, CT should be incorporated into a multimodal cardiovascular imaging pathway for the assessment of aortic valve replacement and for guiding clinical management. Today, in several centers, it is currently the test of choice after the initial echocardiographic assessment.

## 3. Prosthetic Paravalvular Leak

**Figure 3:**
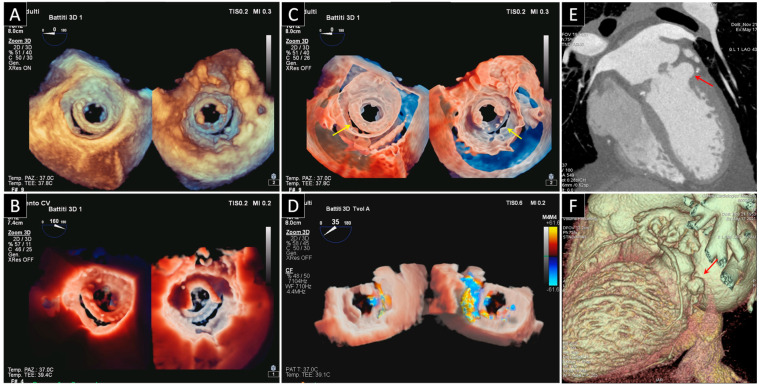
Bioprosthetic mitral valve detachment plus annulus pseudoaneurysm. (**A**) Standard 3D TEE rendering of prosthetic mitral valve from left atrial (surgeon’s view) and ventricular perspective demonstrating detachment from 6 to 9 o’clock due to prosthesis dehiscence. (**B**) Transillumination rendering technique with virtual light source highlighting the localization of the detachment. (**C**) 3D transparency technique more clearly delineating the borders and edges of the paravalvular defect (yellow arrows). (**D**) 3D transparency feature merged with color clearly showing the presence of a severe regurgitation through the area of periprosthetic detachment. (**E**) CT imaging revealing a mitral prosthetic annulus pseudoaneurysm (red arrow) at the ventricular posterolateral wall just under the sewing ring near the detachment of the prosthetic valve in the same patient (**F**) 3D volumetric CT reconstruction of the pseudoaneurysm (red arrow).

PVL is defined as a paraprosthetic regurgitation with a free space close to the sewing ring (Figure 3). The incidence of PVL is reported to be as high as 20% in surgical prosthesis, however the presence of clinically significant PVL needing repair is much lower, ranging from 1 to 5% of PV. PVL is a common complication of transcatheter aortic valve replacement, especially in patients with a bicuspid aortic valve, and its rate is found to be consistently higher (moderate to severe PVL varies from 0% to 24%, mild PVL from 7% to 70%) than after surgical aortic valve replacement [22]. PVLs are usually correlated to annular calcification, tissue friability, infections (as endocarditis) or technical factors [23] Currently, according to the American and European guidelines [11,24], surgical repair is the standard treatment for patients with heart failure or intractable hemolysis due to PVLs. On the other hand, surgical intervention is associated with higher mortality rates than in the first PV implantation (6–14%) [25]. As a result of this augmented risk, there is presently a great interest in percutaneous procedures [26]. Not all patients are eligible for percutaneous closure, their selection is of the utmost importance, and 3D TEE is an ideal technique for carrying out an accurate determination of the anatomic characteristics of PVLs for precisely assessing the site, and for determining the size and shape of single or multiple leaks.

### 3.1. Mitral Paravalvular Leak

**Figure 4:**
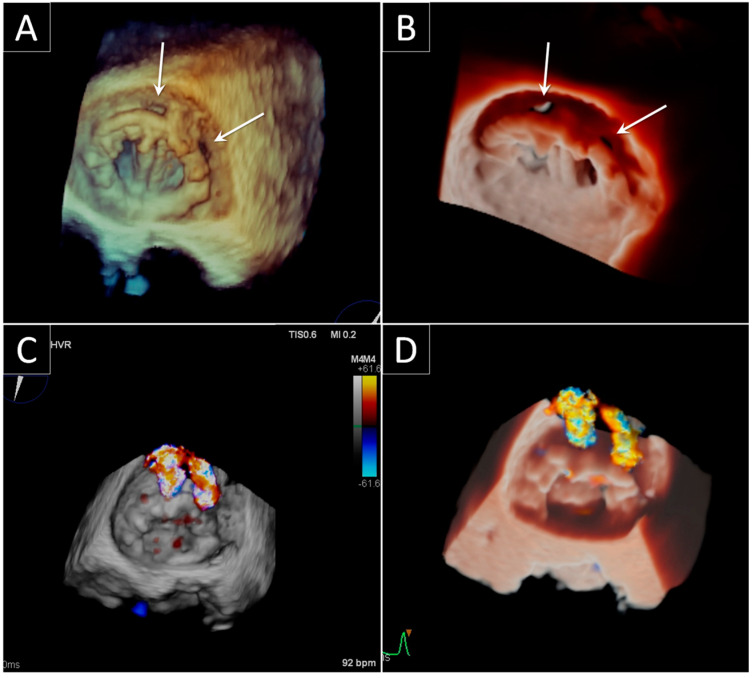
Bileaflet mechanical mitral paravalvular leak. (**A**) Standard 3D TEE rendering and (**B**) transillumination technique of the bioprosthetic mitral valve from atrial perspective showing two paravalvular leaks (arrows) at the prosthetic anterior hinge point and at 2 o’clock. (**C**) Standard 3D TEE rendering and (**D**) transillumination merged with 3D color demonstrating the two jets of regurgitation throughout the paravalvular leaks.

Dyspnea, heart failure associated to pulmonary hypertension, and hemolysis are the classical clinical presentations that should lead you to think of a significant mitral PVL. TTE is once again the first line of examination, yet it is often limited in the mitral PVL assessment, and requires the use of multiple transducer positions, including off axis views. Detection of regurgitation with TTE is more difficult for PV in the mitral positions, particularly in mechanical valves, as a result of shielding and reverberations of the PV. The same principles and methods used for quantification of native valvular regurgitation can also be used for PV, even if with lower effectiveness. Luckily, the presence of significant regurgitation can be suggested from indirect clues coming from various Doppler-parameters. These clues include a normal PHT (<130 ms) associated with parameters that show an increase in transprosthetic flow (Ewave > 200 m/s and ΔP_mean_ > 6 mmHg), particularly when the high flow is not proportional to the systemic flow ejected (i.e., when DVI > 2.2). The presence of these findings in a patient with symptoms of heart failure or hemolysis, represents a clear indication for TEE [1,27]. In fact, TEE has been considered the gold standard for mitral PV assessment and for mitral PVL diagnosis. One of the main reasons for TEE being the gold standard lies in the excellent visualization of the atrial side of the mitral prosthesis enabled by the posterior position of the transducer that makes it possible to avoid reverberation artifacts and acoustic shadowing that cover the ventricular side. A mitral PVL is diagnosed when a regurgitant jet originating from echoes dropout between the outer margin of the prosthetic ring and the native annular tissue is visible at color Doppler acquisition. Once the PVL has been detected, next in line is an assessment of the site of the defect and the severity of its regurgitation. The site can be determined using neighboring anatomic landmarks such as atrial septum, aortic valve or left appendage. The severity of regurgitation can be assessed by the same criteria used to define the severity of native valvular regurgitation. Thanks to the technological improvement of the 3D TEE, an increasingly realistic imaging of the leaflets of mechanical and biological prostheses has been provided in recent years. One of the advantages of this rapidly evolving technique is that it allows visualization from both the atrial and ventricular perspective. 3D TEE also allows us to get a panoramic view of the suture ring, leaflets and discs and may also show the exact number, site, size, and shape (circular, linear, crescent, or irregular) of the PVLs. The 3D TEE data should be presented in the surgical view, with the aortic valve at the top (12 o’clock) and the left appendage at approximately the 9 o’clock position. According to this virtual clock, the localization of the PVLs should be reported as a single hour or as a range of hours [28,29]. The latest developments of the photorealistic 3D rendering make it even easier to interpret the features of the PVL and to reach a better overall understanding of the PVD. One of these post-processing imaging tools, the trans-illumination, makes it possible to change the lightening conditions and therefore to improve contrast, to change shadows and to add more depth perception. Moreover, the addition of color to Doppler evaluations allows us to visualize the flow trajectory, while transparency peels away layers to see the flow origin (Figure 4). Another important advantage of the conventional 3D TEE rendering is the possibility of measuring the proper dimensions of the defect through a system of perpendicular planes crossing the leak, and this can help the interventional cardiologist in choosing the best device for the percutaneous closure. Last, 3D TEE allows for real-time imaging of the catheters within the heart, thus facilitating the maneuvers of the interventionist and the correct positioning of the occluders during the procedure of percutaneous closure.

### 3.2. Aortic Paravalvular Leak

Small aortic PVLs are not uncommon, especially during perioperative examination early after surgery. The majority of these leaks, however, are clinically and hemodynamically insignificant [30]. Significant aortic PVLs, causing heart failure or hemolysis, are more common in correspondence of the non-coronary cusp [23].

TTE is useful in identifying the aortic PVL, particularly when PVL is in the anterior position as the posterior region is hidden by reverberation produced by the prosthetic annulus. Generally, acoustic shadowing caused by the loss of ultrasound penetration beyond prosthesis may limit the evaluation of mitral PV, however it is less of an issue for the identification of prosthetic aortic regurgitation. The optimal views for the detection of regurgitant jets include the parasternal long-axis, parasternal short axis, apical long-axis, and 5-chamber views. In the parasternal short-axis view, color flow Doppler interrogation of the sewing ring may be able to localize and define the extent of a PVL. However, in this view, acoustic shadowing may obscure jets in the region of the non-coronary sinus [1]. TTE is also used to represent the aortic PVLs in a clockwise fashion [31]. This is a very useful and simple system to follow-up the PVL, however the TTE o’clock representation of the Valsalva aortic sinus does not correspond to TEE or to the surgical view representation and, as such, using this representation, PVL can be misleading when the patient goes to TEE or surgery.

Fluoroscopy is very useful for clarifying an intra versus PVL regurgitation in the bileaflet PV. In fact, in case of a regurgitation of unknown origin, normal closing and opening prosthetic angles can exclude the case of intra-prosthetic regurgitation and lead us to the PVL diagnosis [10].

As previously described, the size of the PV and its position in relation to the transducer make 2D and 3D TEE less effective in assessing the PV in aortic position than in mitral position. TEE is very useful in detecting a PVL in a posterior position (corresponding to the non-coronary sinus), as this region is closer to the transducer in contrast with the anterior region, hidden by reverberation produced by the annulus. Furthermore, it is recommended to display 3D color Doppler data together with 3D TEE anatomic information to clearly show site, size and shape of the aortic leaks using an “en face” view and also, by rotating the volumetric dataset, a ventricular perspective.

Once again, CT images, which can be reconstructed in any desired imaging plane after acquisition, could provide an accurate definition of PVL due to endocarditis and root abscess and their relationship with the Valsalva sinus and the coronary artery [13,20,21].

## 4. Bioprosthetic Structural Valve Degeneration

SVD is an acquired intrinsic bioprosthetic valve abnormality defined as deterioration of the leaflets or supporting structures resulting in thickening, calcification, tearing, or disruption of the PV materials with eventual associated valve hemodynamic dysfunction, manifested as stenosis, regurgitation or a combination of stenosis and regurgitation [32]. Bovine pericardial valves have a greater propensity to develop stenosis, whereas porcine valves have a tendency to develop leaflet tear with regurgitation [33]. Time-related SVD is the major drawback of bioprosthetic valves. Leaflet tissue deterioration, whether calcific or noncalcific, is the major cause of bioprosthetic valve failure and is associated with several risk factors; the most common of these risks include young patient age, renal failure, abnormal calcium metabolism, and prosthesis-patient mismatch of the implanted valve [34,35]. Valve-in-valve procedures have been identified as a feasible, less-invasive treatment option for patients with degenerated surgically implanted bioprostheses, and the ACC/AHA guidelines currently recommend this approach in high-risk patients with aortic bioprosthesis dysfunction [11,24]. In these patients, valve-in-valve procedures have been associated with a high rate (95%) of successful valve implantation and a mean 30-day mortality of 8% [36].

### 4.1. Mitral Bioprosthetic SVD

**Figure 5:**
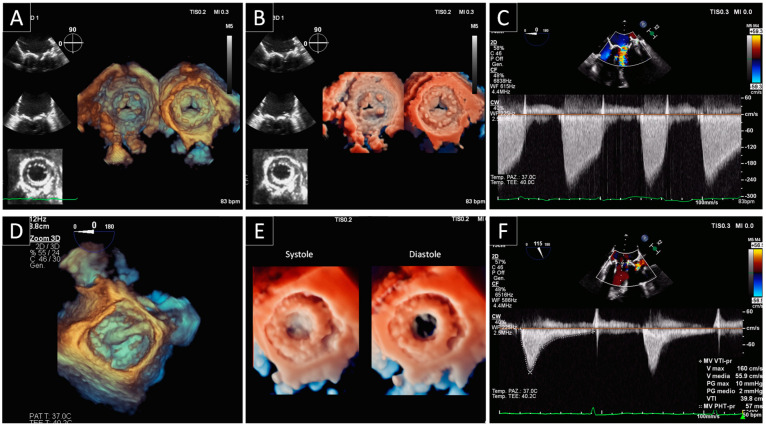
SVD of a biological prosthesis in mitral position treated with valve-in-valve procedure. (**A**) Standard 2D and 3D TEE rendering and (**B**) 3D transillumination technique of a bioprosthetic mitral valve from atrial and ventricular perspective demonstrating leaflets thickening and immobility. The SVD causes incomplete opening of two of the cusps and therefore results in a reduced anatomic and effective orifice area. (**C**) Increased transprosthetic gradient and pathological PHT at continuous wave Doppler by TEE. (**D**) Transseptal positioning of the transcatheter PV displayed from the left atrium using 3D TEE rendering during the valve-in-valve procedure. (**E**) Fully deployed mitral valve-in-valve in systole and diastole depicted using transillumination technique. (**F**) Normalization of transprosthetic gradient and PHT at continuous wave Doppler by TEE.

Similar to what we have seen in regard to the aortic valves, and also in the context of a failing mitral bioprosthetic valve, it is essential to assess early morphological leaflet changes associated with hemodynamic dysfunction (stenosis or regurgitation). In presence of severe, symptomatic stenosis or regurgitation, the patient should be referred to surgery or to mitral valve-in-valve implantation (Figure 5).

2D and Doppler TTE should be targeted to the anatomy of the bioprosthetic valve and to both flow-dependent and flow-independent Doppler parameters (ΔP_mean_, E wave, PHT and DVI) indicating severe hemodynamic dysfunction.

Whenever a mitral valve-in-valve procedure is proposed, 3D TEE is needed to determine the internal dimensions of surgical mitral PV and therefore to choose the optimal size of the transcatheter aortic PV to be implanted. When the true internal diameter falls between two recommended transcatheter sizes, the smaller one can be used if the valve is stenotic. The larger one should be used to decrease the risk of embolization if the valve is regurgitant. In valve-in-valve procedures, the risk of left ventricular outflow (LVOT) obstruction is very low as in most cases the anterior leaflet of the mitral valve is absent. To further mitigate the risk of LVOT obstruction, it is mandatory to measure the aorto-mitral-annular angle (>115°) and the height of the surgical prosthesis. Moreover, it is important to remember that the stent frames of pericardial leaflets are taller than porcine bioprosthetic valves and cover the stent fully, while porcine leaflets are shorter and proved less cover for the transcatheter stent frame. This translates into less risk of LVOT obstruction with valve-in-valve with porcine surgical bioprosthesis [37].

CT is strongly recommended for the planning of a transcatheter mitral valve-in-valve procedure. Measurements of the surgical PV need to be taken along inner border, taking care to optimize image acquisition and image reconstruction, using thin slices without cardiac motion, and wide grayscale display settings to minimize beam hardening and blooming artifacts [38]. Compared to echocardiography, CT achieves a more accurate measurement of the aorto-mitral-annular angle as well as of the height of the surgical prosthesis and remains crucial for procedural success thanks to its capability for accurate modelling of the baseline LVOT and post-procedure neo-LVOT [37,38].

### 4.2. Aortic Bioprosthetic SVD

In routine practice, TTE is the primary imaging modality for the assessment of aortic bioprosthetic SVD. Quantitative hemodynamics parameters, as assessed by Doppler TTE, depend on the type and size of PV and the native valvular anatomy. Therefore, a comparison of the baseline with the follow-up hemodynamic profile of the specific valve in the specific patient is recommended for evaluating SVD [32].

Recently, Dvir et al. and Salaun et al. [32,39] proposed the categorization of SVD in different stages of severity, each associated with a specifically recommended clinical approach. Stage 1 corresponds to early morphological leaflet changes, without hemodynamic sequelae. This is present when TTE or TEE demonstrates abnormal leaflet thickness, increased echogenicity suggestive of fibrosis or calcification, or abnormal mobility. Doppler evaluation in Stage 1 SVD shows normal hemodynamic parameters (V_max_ < 3 m/s; ΔP_mean_ < 20 mmHg with an increase in mean gradient during follow-up < 10 mmHg; DVI > 0.35; AT < 100 ms; AT/ET < 0.32; EOA > 1.2 cm^2^ for BSA > 1.6 m^2^ and >1 cm^2^ for BSA < 1.6 m^2^). Stage 2 refers to morphological abnormalities of valve leaflets associated with hemodynamic dysfunction (moderate stenosis or regurgitation). This is defined at Doppler evaluation by hemodynamic parameters of moderate stenosis (V_max_ 3–4 m/s; ΔP_mean_ 20–40 mmHg, associated to an increase in mean gradient during follow-up of 10–20 mmHg; DVI 0.25–0.35; AT 80–100 ms; AT/ET 0.32–0.37; EOA 1–1.2 cm^2^ for BSA > 1.6 m^2^ and EOA 0.8–1.1 cm^2^ for BSA < 1.6 m^2^) or moderate regurgitation. Stage 3 refers to the development of severe stenosis or regurgitation. This is defined at Doppler evaluation by hemodynamic parameters of severe stenosis (V_max_ > 4 m/s; ΔP_mean_ > 40 mmHg associated to an increase in mean gradient during follow-up > 20 mmHg; AT > 100 ms; AT/ET > 0.37; EOA < 1 cm^2^ for BSA > 1.6 m^2^ and EOA < 0.8 cm^2^ for BSA < 1.6 m^2^), or severe prosthetic regurgitation. Stage 3 SVD valves should be approached with a re-intervention whenever the patient is symptomatic.

The higher resolution makes TEE an even more helpful imaging technique for evaluating and identifying the mechanism of biological SVD (leaflet thickening/calcification or leaflet flail or tear) [35]. Since the predominant mechanism of biological prosthetic dysfunction lies in the valve’s structural deterioration, the role of TEE is essential in assessing aortic insufficiency and in distinguishing between transvalvular and paravalvular regurgitation. The 3D “en face” surgical view of the valve is very useful for determining the presence, origin, direction, and extension of regurgitant jets.

Bioprosthetic valve thrombosis, once considered a relatively rare clinical entity, is now (thanks to transcatheter aortic valve replacement) acknowledged as an important cause of valve dysfunction [40]. The advent of transcatheter procedures with the recent findings of subclinical leaflet thrombosis at CT, has generated enormous interest, casting new light on its true incidence and clinical relevance in both transcatheter and surgically implanted bioprosthetic valves. TEE is extremely useful in thrombus identification, which is more commonly seen on the downstream aspect of the valve that is the arterial side for aortic bioprosthetic valve.

CT allows the accurate visualization of valve anatomy and it is particularly helpful for the detection of valve thrombosis and pannus formation. Specific CT visualizations include stent frame expansion and eccentricity index, degree of leaflet/s thickening or hypoattenuation [41], motion reduction and calcification [42]. CT is therefore essential and should be performed systematically to assess bioprosthetic SVD. However, it is important to note that CT cannot determine aortic valve gradients and that TTE is necessarily, even now, the first modality imaging when thrombosis or pannus is suspected.

## 5. Prosthetic Endocarditis

**Figure 6:**
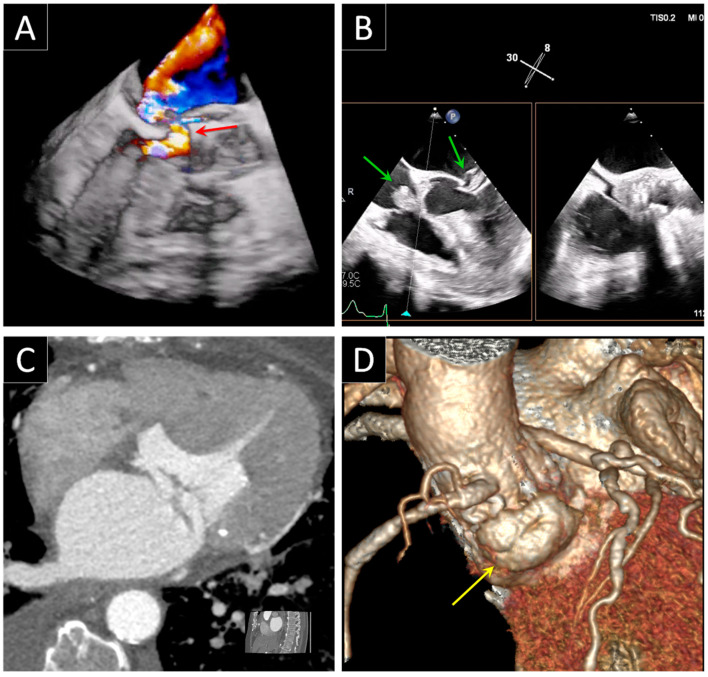
Bioprosthetic aortic valve abscess. (**A**) Standard 3D TEE merged with color demonstrating the presence of the PVL associated with the disruption of the mitral-aortic junction (red arrow) in a patient with infective endocarditis. (**B**) Vegetations are clearly visible on the tricuspid valve and mitral valve leaflets (green arrows) using 2D TEE x-plane view (**C**) CT image and (**D**) 3D CT reconstruction showing the abscess (yellow arrow) at the level of mitral-aortic junction.

**Figure 7:**
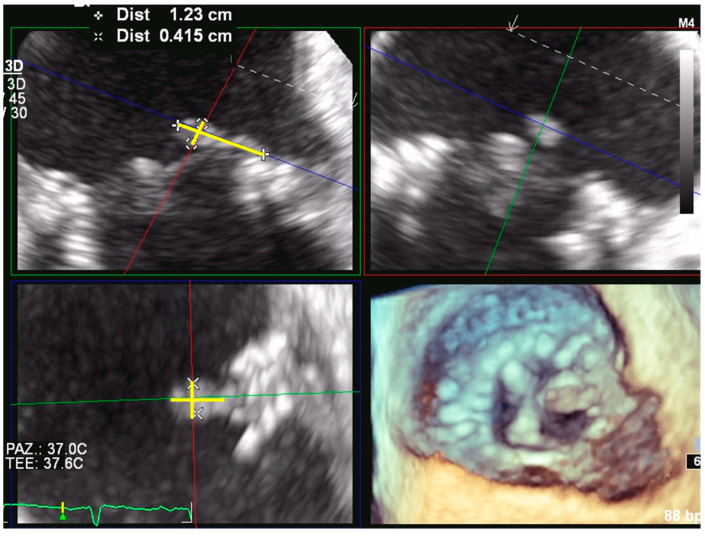
Bioprosthetic mitral valve endocarditis. Multiplanar reconstruction allows proper alignment of the planes perpendicular to the long and short axis of the mobile vegetation at the left atrial side of the mitral prosthesis. This approach ensures we have more reliable 3D measurements of vegetation’s dimensions (yellow lines) that are important for guiding the therapeutic intervention.

PVE is the most severe form of infective endocarditis and occurs in 1–6% of patients with PV. This affects mechanical and biological PV equally [43]. Diagnosis is more difficult in PVE than in native valve endocarditis as clinical presentation is frequently atypical and the Duke criteria has lower sensitivity in the setting of PVE [44]. Post-operative PVE occurs during the first year after valve replacement, frequently caused by staphylococci. The infection usually involves the junction between the sewing ring and the annulus, leading to perivalvular abscess, dehiscence, pseudo-aneurysm and fistulae (Figure 6). After 1 year, the distribution of microorganisms causing PVE is similar to that causing endocarditis on native valve. In late PVE, infection is frequently located on the prosthetic leaflets, leading to vegetations, cusp rupture and perforation [45]. TTE remains the cornerstone of imaging. This is in fact rapid, straightforward, and, in many cases, diagnostic [46]. A negative TTE, though, can be frequently observed in PVE, due to prosthetic reverberations and shadowing. For this reason, TEE imaging is strongly recommended [11,24,45] and, if negative, the procedure must be repeated in case of high suspicion of infective endocarditis. TEE is the gold standard, especially for the detection of vegetations and the measurement of its length, which both have a major impact on the risk of embolism and on the indication of early surgery. TEE is also preferable to TTE in localizing PVL and in the assessment of leaflet malfunction and prosthetic dehiscence. 3D TEE has an even wider role than 2D TEE. In fact, 3D TEE provides incremental anatomic information allowing for a far better visualization of cardiac anatomy. Therefore, it helps in assessing paravalvular’s extension of infection, prosthetic dehiscence, valve perforation and in measuring the diameter of irregular masses (i.e., vegetations) (Figure 7). Since the major diameter of vegetations is currently the cutoff point to indicate surgery in patients without other surgical indications, differences between measurements by 2D and 3D TEE may have therapeutic consequences [47].

CT has excellent spatial resolution enabling visualization of paravalvular complications such as abscesses or aneurysms and has potentially less imaging artifacts from the PV than TEE does [48,49]. However, it is less sensitive than TEE in detecting small vegetations. CT may also reveal concomitant pulmonary disease, may identify splenic and other abscesses and allows a complete visualization of the intracranial vascular tree.

Last, nuclear molecular techniques have an increasing role for patients with suspected PVE and diagnostic difficulties. Radiolabeled leukocyte scintigraphy or 18-F-fluorodeoxyglucose positron emission tomographic–computed tomographic (FDG-PET/CT) scanning can be helpful in detecting peripheral emboli and cardiac or extracardiac sites of infection. The current guidelines have suggested that adding FDG-PET/CT findings as an additional major criterion to the modified Duke criteria increased the sensitivity without compromising specificity by reclassifying possible diagnoses to definite infective endocarditis [11,24,50,51]. On the other hand, positive uptake visualized by PET/CT is not specific and can be observed in tumors and any type of inflammatory disease and non-pathological uptake might be observed after PV replacement, not only during the first months but also years after surgery [52]. Radiolabelled leukocyte scintigraphy is less sensitive but more specific than PET–CT for the diagnosis of prosthetic-valve IE. This technique is particularly useful during the first three months after cardiac surgery, when PET/CT might lead to false-positive findings [45]. The limited availability of radiolabelled leukocyte scintigraphy explains why it is not used more commonly to confirm the diagnosis of PVE when it remains doubtful after other imaging techniques, including PET/CT.

## 6. Conclusions

The epidemiology and management of PVD are both in continuous evolution. Guidelines provide specific recommendations about their management, however, careful attention to individual patient characteristics, type of dysfunction, and risk of PVE sequelae must be considered when making therapeutic decisions.
